# Supplementary material for: Stage Shifting by Modifying the Determinants of Breast Cancer Stage at Diagnosis: A Simulation Study
Source: Cancers (Basel). 2024 Mar 19;16(6):1201. doi: 10.3390/cancers16061201 (PMC10969221; doi:10.3390/cancers16061201)
Supplement: Supplementary file 1 [file cancers-16-01201-s001.zip › Supplementary Figure S1.pdf]

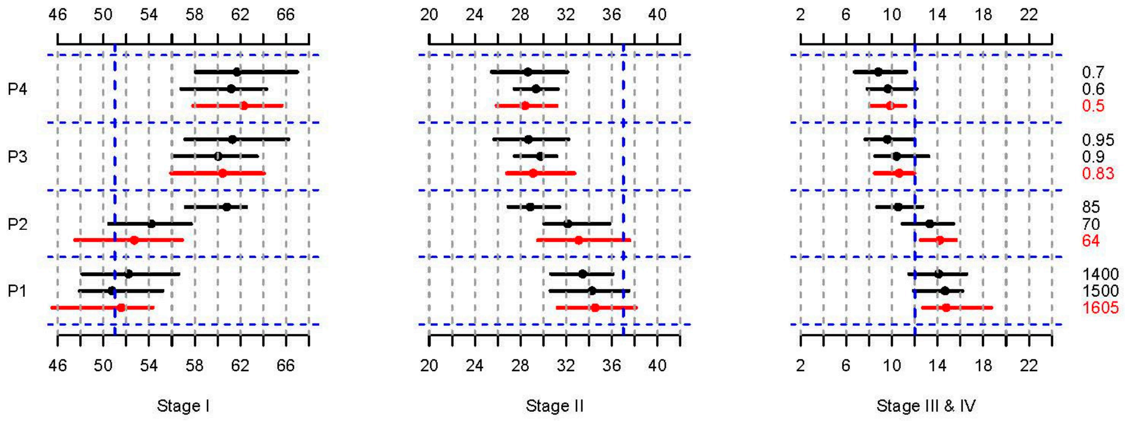

Point estimates (medians – red and black dots) and 95% percentile intervals ((PIs), red and black lines) of the observed (red) and predicted (black) stages of the breast cancer, respectively, after modifying the means and proportions of the policy target factors from Scenario I. Cumulative effects are shown based on modifying the distributions of the factors from Designs P1 to P4 (See Table S2 for the variable names). The blocks between blue horizontal dotted lines denote the effects for each factor. The vertical dot lines represent 2% while the vertical blue dashed lines represent the percentage of women diagnosed in that stage in the real data set.

Figure S1: Impact from modifying the distributions for policy target factors from Scenario I.
