# Supplementary material for: Stage Shifting by Modifying the Determinants of Breast Cancer Stage at Diagnosis: A Simulation Study
Source: Cancers (Basel). 2024 Mar 19;16(6):1201. doi: 10.3390/cancers16061201 (PMC10969221; doi:10.3390/cancers16061201)
Supplement: Supplementary file 1 [file cancers-16-01201-s001.zip › Supplementary Table S1.pdf]

Table S1. Data Dictionary.

**Variables**	**Description**	**Coding**
Total dietary energy intake	Baseline total dietary kcal intake	Continuous.
Total dietary protein intake	Daily protein intake in g/day	Continuous
Ever been pregnant	Have you ever been pregnant?	1: yes; 0: no
Spend in the sun 11 am-4 pm, June-August	During this past June through August, on a typical day outdoors, approximately how much time did you spend in the sun between 11am and 4pm?	1: more than 1 hour per day, 0: less than 1 hour.
Current stressful situations	Stress: Is your current situation one of ?<br>1) Your work around the home is not appreciated.<br>2) Your friends are a bad influence.<br>3) You would like to move but can't.<br>4) Your neighborhood or community is too noisy or polluted.<br>5) You have a parent, a child or a partner who is in very bad health and may die.<br>6) Someone in your family has an alcohol, drug, or gambling problem.<br>7) People are too critical of you or what you do.	1: yes; 0: no
Birth control pills use	Have you ever taken birth control pills for any reason? Do not include birth control	1:yes; 0: no
