# Supplementary material for: Stage Shifting by Modifying the Determinants of Breast Cancer Stage at Diagnosis: A Simulation Study
Source: Cancers (Basel). 2024 Mar 19;16(6):1201. doi: 10.3390/cancers16061201 (PMC10969221; doi:10.3390/cancers16061201)
Supplement: Supplementary file 1 [file cancers-16-01201-s001.zip › Supplementary Table S2.pdf]

Table S2. Modified distributions of lifestyle and health factors for policy targets.

Design	Factor	Modified distribution	Effect
P1	Total dietary energy intake (kcal/day)	(**<u>1605</u>**, 1500, 1400)	Increased Risk
P2	Total dietary protein intake (g/day)	(**<u>64.0</u>**, 70.0, 85.0)	Decreased Risk
P3	Ever had a mammogram	(**<u>0.83</u>**, 0.90, 0.95)	Decreased Risk
P4	Spend time in the sun 11 am-4 pm, June-August	(**<u>0.50</u>**, 0.60, 0.70)	Decreased Risk

These factors were based on results from Scenario I that shifted the stage at diagnosis downwards to Stage I. The modified distributions were considered based on one and two units shift (black) up or down from the observed (bold, underlined) distributions of the ATP data.
