# Supplementary material for: Stage Shifting by Modifying the Determinants of Breast Cancer Stage at Diagnosis: A Simulation Study
Source: Cancers (Basel). 2024 Mar 19;16(6):1201. doi: 10.3390/cancers16061201 (PMC10969221; doi:10.3390/cancers16061201)
Supplement: Supplementary file 1 [file cancers-16-01201-s001.zip › Supplementary Table S3.pdf]

Table S3. Comparison of summary statistics between the observed data and simulated data for all of the variables included in the final multivariable partial proportional odds regression model for the real data analysis.

Variables	Summary Statistics			
	Observed data (sample size = 492)		Simulated data (sample Size = 1000)	
**Continuous variables**	**Mean**	**SD**	**Mean**	**SD**
Age at cancer diagnosis (years)	59.77	9.93	60.53	9.6
Time to health center (minutes in log scale)	1.96	0.94	1.2	0.73
Elixhauser comorbidity index	0.88	1.13	0.78	0.93
Total dietary energy intake* (kcal/day)	1605.46	576.53	1605.5	561.04
Total dietary protein intake* (g/day)	63.76	25.04	64.41	24.41
**Binary variables**	**N**	**%**	**N**	**%**
Family history of breast cancer (yes)	89	18.01	177	17.7
Ever been pregnant* (yes)	444	90.2	902	90.2
Birth control pills use* (yes)	427	86.8	877	87.7
Ever had mammogram* (yes)	409	83.1	829	82.9
Smoked at least 100 cigarettes in your life? (yes)	280	57	543	54.3
Spend in the sun 11 a.m. to 4 p.m., June – August* (< 1 hour)	248	50.4	504	50.4
Current stressful situations* (≥ 1)	263	53.5	527	52.7
**Categorical variables**	**N**	**%**	**N**	**%**
Household income				
< $50 K	192	39	384	38.4
$50 - $100 K	201	41	426	42.6
> $100 K	99	20	190	19
Cancer stages*				
Stage I	253	51.4	524	52.4
Stage II	180	36.6	334	33.4
Stages III & IV	59	12	142	14.2

* included in simulation study
