# Supplementary material for: Stage Shifting by Modifying the Determinants of Breast Cancer Stage at Diagnosis: A Simulation Study
Source: Cancers (Basel). 2024 Mar 19;16(6):1201. doi: 10.3390/cancers16061201 (PMC10969221; doi:10.3390/cancers16061201)
Supplement: Supplementary file 1 [file cancers-16-01201-s001.zip › Supplementary Table S4.pdf]

R code for simulation study
#Program name: predict_y
Program description: R code to calculate log odds cancer stages and convert to the probability
# of diagnosing in the stages I, II and III & IV using inv_logit function and finally the proportion #of stages of the cancer.

Inputs a simulated data matrix (simulatedData) with exactly 14 columns (screened by #Wang et al, 2022*) and the values of the regression parameters estimated by Wang et al, 2022.

Output is proportion of the predictive stages based on the probability of diagnosing in the #stages I, II and III & IV using inv_logit function.

Project name: Catching cancer when it is small
Project Scientists: Gyandendra Pokharel and Karen Kopciuk
Author: written by Gyanendra Pokharel, Winnipeg
Initial creation date: August 20198, last edit: March 2023
Written for R version 4.2.2, Operating system: any

Here are parts of this function code which shows sequential structure, line and space delimiters, and
descriptions of input/output for a step

```
# Logit function


inv_logit <- function(logOdds){ #Input: log odds and output: stage
 probability
  pi <- exp(logOdds)/(1 + exp(logOdds))
  return(pi)
}

# Simulate the response (proportions of breast cancer stages)

predict.y <-function(betaPO,betaPPO,simulatedData,beta0,sig){

#initialize the stage probabilities.

  pi1 =pi2to3=0.0
  pi2=pi3to3 =0.0
  pi3=0.0

#create a matrix of parameter values

  beta00 <-data.frame(matrix(c("beta02","beta03",beta0),nrow=2,byrow=F))
  colnames(beta00) <- c("Variables","estimates")
  beta <-data.frame(rbind(betaPO,beta00))

#extract variables satisfying PO and PPO assumptions from the simulated
 input.

  dataPO <- simulatedData[,match(betaPO$Variables,colnames(simulatedData))]
  dataPPO <- simulatedData[,match(betaPPO$Variables,colnames(simulatedData))]
```

```
# calculate the contribution of input variables and parameters for both PO
 and PPO assumptions

  covariatePO <- as.numeric(t(betaPO[,2]))%*%t(dataPO)
  covariatePPO1 <- as.numeric(t(betaPPO[,2]))%*%t(dataPPO)
  covariatePPO2 <- as.numeric(t(betaPPO[,3]))%*%t(dataPPO)

# Sample random error from normal distribution with mean 0 and standard
 devaiation sigma.

  noise<-rnorm(1,0,sig)

# Calculate log odds

  logOdds1 <- (as.numeric(beta0[1])+(covariatePO+covariatePPO1+noise))
  logOdds2 <- (as.numeric(beta0[2])+(covariatePO+covariatePPO2+noise))

# Calculate cumulative probabilities using inv_logit() function defined
# above

  pi2to3 <-inv_logit(logOdds1)
  pi3to3 <-inv_logit(logOdds2)

# Calculate individual stage probabilities

  pi1 <-1-pi2to3
  pi2 <-pi2to3-pi3to3
  pi3 <-pi3to3

#Sample stages from (1,2,3) using the probability of stages.

  y <- c()
  for (i in 1:nrow(simulatedData)){
      y[i] <- sample(
        x = c(1,2,3),
        size = 1,
        replace=T,
        prob = c(pi1[i], pi2[i], pi3[i]))
  }

# Find the number of individuals in each stages and convert to the
 proportion.
  n.y <- table(y)
  p.y <-table(y)/nrow(simulatedData)

# cbind and return parameter values,individual stages, and predictive stages.

  predict<-cbind(n.y,p.y)
  data.out <-list(beta,y,predict)
  return(data.out)
}
```

[*]Wang Q, Aktary ML, Spinelli JJ, Shack L, Robson PJ, Kopciuk KA. Pre-diagnosis lifestyle, health history and psychosocial factors associated with stage at breast cancer diagnosis – Potential targets to shift stage earlier. Cancer Epidemiology. 2022;78:102152.
